# Isolated REM sleep behaviour disorder: current diagnostic procedures and emerging new technologies

**DOI:** 10.1007/s00415-022-11213-9

**Published:** 2022-06-24

**Authors:** Samantha Bramich, Anna King, Maneesh Kuruvilla, Sharon L. Naismith, Alastair Noyce, Jane Alty

**Affiliations:** 1 Wicking Dementia Research and Education Centre, University of Tasmania, Hobart, Australia; 2 Neurology Department, Royal Hobart Hospital, Hobart, Australia; 3 Charles Perkins Centre and Brain and Mind Centre, The University of Sydney, Sydney, Australia; 4 Wolfson Institute of Population Health, Queen Mary University of London, London, UK

**Keywords:** REM sleep behaviour disorder (RBD), Diagnostic methods, Sleep disorders, Neurology, Parkinson’s disease, Dementia

## Abstract

Isolated REM sleep behaviour disorder (iRBD) is characterised by dream enactment behaviours, such as kicking and punching while asleep, and vivid/violent dreams. It is now acknowledged as a prodromal phase of neurodegenerative disease—approximately 80% of people with iRBD will develop dementia with Lewy Bodies, Parkinson’s disease or another degenerative brain disease within 10 years. It is important that neurologists and other clinicians understand how to make an early accurate diagnosis of iRBD so that affected people can have the opportunity to take part in clinical trials. However, making a diagnosis can be clinically challenging due to a variety of reasons, including delayed referral, symptom overlap with other disorders, and uncertainty about how to confirm a diagnosis. Several methods of assessment are available, such as clinical interview, screening questionnaires and video polysomnography or ‘sleep study’. This review aims to support clinical neurologists in assessing people who present with symptoms suggestive of iRBD. We describe the usefulness and limitations of each diagnostic method currently available in clinical practice, and present recent research on the utility of new wearable technologies to assist with iRBD diagnosis, which may offer a more practical assessment method for clinicians. This review highlights the importance of thorough clinical investigation when patients present with suspected iRBD and emphasises the need for easier access to diagnostic procedures for accurate and early diagnosis.

## Introduction

REM sleep behaviour disorder (RBD) is a sleep disorder in which people physically act out their dreams. During sleep, the body cycles through four stages: N1, N2, and N3, which are NREM (non-rapid-eye movement) stages, and rapid-eye movement sleep, or REM sleep. Normally each sleep cycle begins in NREM sleep, starting in N1 and transitioning through to the progressively deeper stages of N2 and then N3, before finally reaching REM sleep. Each cycle lasts for approximately 90 min and tends to occur between 3 and 5 times per night [1]. In general, during REM sleep all skeletal muscle tone (except for the eye muscles) is inhibited so that the body is unable to move. However, in RBD the brain mechanisms underlying this atonia are impaired due to dysfunction in the systems that produce REM sleep paralysis [2]. People with RBD experience vivid and violent dreams, and the acting out of these dreams (such as shouting, punching and kicking movements), which they are often unaware of, can pose a risk to both the person with RBD as well as their bed partner [3]. RBD can occur with several other conditions [4] but can also exist independently in otherwise healthy individuals this is known as isolated or idiopathic RBD (iRBD). Studies have shown that approximately 80% of individuals with iRBD will develop a neurodegenerative disease (ND), such as dementia with Lewy bodies and Parkinson’s disease (PD) within 10 years of first diagnosis [5, 6], making it a prodrome of early ND and probably an early stage synucleinopathy in its own right [7]. It is, therefore, important for clinicians to identify individuals with iRBD as this offers an opportunity for early recruitment into clinical trials and observational studies, which aim to follow patients with iRBD over time to identify risk factors for ND development, as well as investigating iRBD genotyping and potential treatment options. Early detection of iRBD can also assist neurologists in their review and management of early stage ND diagnosis.

The International Classification of Sleep Disorders (ICSD-3) states the following diagnostic criteria for REM sleep behaviour disorder (RBD):

“*Repeated episodes of sleep-related vocalization and/or complex motor behaviours; these behaviours are documented by polysomnography (PSG) to occur during REM sleep or based on clinical history of dream enactment, are presumed to occur during REM sleep; PSG recording demonstrates REM sleep without atonia (RSWA); and the disturbance is not better explained by another sleep disorder, mental disorder, medication or substance abuse*”

American Academy of Sleep Medicine (2014) International classification of sleep disorders, 3rd edn. American Academy of Sleep Medicine, Darien [8]

The International RBD Study Group (IRBDSG) also recently published definitive guidelines for RBD diagnosis stating that video PSG (vPSG) is mandatory for the identification of iRBD, and must find either isolated RSWA or motor events (i.e., any type of movement in sleep captured on video) [9]. Thus, for a confirmed diagnosis of iRBD, a documented history of dream enactment behaviour, along with a full vPSG to obtain evidence of RSWA or motor events during sleep is required. This is problematic for many, if not most, clinicians as unfortunately, access to vPSG is limited due to expense and/or is unavailable in many locations throughout the world. Availability of sleep specialists and overnight bed use in hospitals is low world-wide, and costs to patients and/or healthcare systems tend to be excessive, causing high discrepancy between capacity and demand [10]. In lieu of a vPSG, several non-diagnostic iRBD screening questionnaires are used, which show varying rates of reliability and validity, and depend strongly on a person’s awareness of their symptoms [11–14]. More recently, new and wearable technology (such as the actigraphy watch) has been developed, which may improve diagnostic accessibility and lower the cost of sleep investigations [15]. An extensive literature search for this narrative review was conducted between October and December 2021, with a focus on papers published from 2011 to 2021. Databases searched included Scopus, Web of Science and PubMed, with numerous search terms, such as “REM sleep behaviour disorder”, “diagnosis”, “diagnostic procedures”, “screening questionnaires”, “clinical interview”, “actigraphy”, and “technology”. The aim of this review is to support clinicians in making a diagnosis of iRBD by outlining the tools currently available in the diagnosis of iRBD, the efficacy of each, and a potential way forward using new technologies for easier access to diagnosis.

## Suspected iRBD

The initial suspicion of iRBD is usually raised by a bed partner, who has noticed unusual behaviours during sleep. These often include yelling, kicking, punching and more complex actions, such as moving items around the room [16]. This may prompt an appointment with the individual’s GP for investigation, but can also be left unattended for many years, especially if iRBD symptoms fluctuate, are considered medically unimportant, or if there is no bed partner at all [17]. Even when presenting in primary care, misdiagnoses may occur due to lack of awareness of iRBD in general medicine and/or symptoms mimicking other common sleep disorders [17, 18]. For example, iRBD body movements may be misdiagnosed as periodic limb movement disorder (PLMD) [19], or obstructive sleep apnoea (OSA) may be misdiagnosed when iRBD presents with frequent nocturnal awakenings [4]. RBD can be associated with prolonged antidepressant use, with certain antidepressant medications found to induce RBD symptoms [20, 21]. In particular, selective serotonin reuptake inhibitors (SSRI’s) have been linked to the onset of RBD symptoms, whereas an association has not been found with tricyclic antidepressant usage [21]. Secondary RBD is also a known feature of multiple system atrophy (MSA) and can occur in multiple sclerosis (MS) and stroke cases [22–25]. However, with secondary RBD, it is important to make a distinction between RBD in the context of synucleinopathies (where it is very frequent in 50% of PD, 80% of DLB, and up to 100% of MSA patients [27]) and other neurological diseases (MS and stroke), where it is a rare manifestation [24, 25]. These similarities with other conditions and associated factors show how initial suspicion of iRBD can be missed, as investigations into uncommon sleep disorders and referral to specialist services (such as neurology or sleep medicine) are rarely considered in primary care settings [17]. Table 1 describes several sleep disorders that are commonly mistaken for iRBD.

**Table 1:** Sleep disorders presenting with similar symptoms to iRBD and clinical evidence that excludes a diagnosis of iRBD

Sleep disorder	Symptoms similar to iRBD	Evidence that excludes iRBD
Obstructive sleep apnoea	Unpleasant dream content Dream enactment behaviours Frequent nocturnal awakenings	No evidence of RSWA Increased apnoea/hypopnea index
Periodic limb movement disorder	Unpleasant dream content Vigorous limb movement during sleep Sleep talking	Vigorous limb movements mainly in NREM sleep Limb movements not solely in REM sleep
NREM parasomnias	Sleep walking Sleep talking Night terrors	No evidence of RSWA
Nightmare disorder	Unpleasant dream content Limb movements during sleep Sleep talking Frequent nocturnal awakenings	No evidence of RSWA

*REM* rapid eye movement, *NREM* non-REM, *RSWA* REM sleep without atonia [2, 19, 26, 28]

Lack of awareness among neurologists and other specialists may also contribute to diagnostic delay, with one study finding that 31% of patients did not receive a timely diagnosis of iRBD (mean delay of 8.7 years from symptom onset) due to failure of their specialist (including neurologists) to recognise the symptoms [17]. Further research shows that even patients presenting with sleep concerns are rarely questioned about specific iRBD symptomology, with the majority of iRBD positive PSG’s being requested for other reasons, such as suspected sleep breathing disorders or insomnia [29]. These findings reveal the importance of thorough clinical assessment when patients present with iRBD symptomology, as lack of initial recognition can hinder the diagnostic process and reduce opportunities for disease management, especially early ND identification and potential treatment.

## Clinical interview

Once initial suspicion of iRBD has been raised and referral made to a Neurology or Sleep Medicine service, thorough history taking, including direct questioning of iRBD symptomatology, is one of the most valuable tools to assist in diagnosis. Enquiring about dream enactment behaviour in the person with suspected iRBD as well as their partner is integral. It is also useful to rule out other possible causes of symptoms, such as other neurological disorders or withdrawal from sedatives or alcohol [30]. There is a paucity of research on the precise predictive value of clinical interview in the diagnosis of iRBD and to date, only one study has elucidated its value. Vignatelli and colleagues investigated inter-observer reliability among trained neurologists who used clinical interview based on ICSD criteria for iRBD diagnosis [31]. Six neurologists examined videotapes of clinical interview with 10 patients experiencing possible iRBD during sleep and were asked to identify the presence of each ICSD criterion for iRBD. Interview questions were based loosely on the ICSD criteria. Inter-rater reliability was high, with overall agreement on iRBD cases at 83%. However, neurologists did note some variance in criteria associated with motor behaviours during sleep, as subjective reports were often unclear. Interestingly, the authors describe that eight of the subjects underwent PSG and iRBD was confirmed in only three of these cases; however, this finding was not discussed in terms of iRBD validation compared to other sleep disorders found on PSG. This limited research shows that clinical interview is useful in identifying potential iRBD cases when based on ICSD criteria; however, vPSG is still necessary to rule out other potential sleep disorders.

Including the patient’s bed partner in the interview process is crucial as many patients with iRBD have no awareness that they act out their dreams during sleep. It is often the bed partner who first notices a change in the affected individual’s sleep when they observe the dream enactment directly or have sustained injuries during the night, such as being kicked or punched. They may also observe numerous unusual activities during sleep, knowing that their partner is not awake, including chasing and throwing behaviours [32], and these observations are vital for assessment, even if they are thought to be unimportant by the patient and their partner [33]. Simple questioning based on the ICSD criteria is often sufficient to elicit this information. The Mayo Sleep Questionnaire (MSQ) developed by Boeve and colleagues, provides a 16-item scale that screens for abnormal sleep behaviours, based solely on bed partner responses; see Table 2 [11]. While this has consistently shown high sensitivity and specificity (100 and 95, respectively) in both healthy control populations as well as those with mild cognitive impairment and Alzheimer’s disease [34], it is still subject to several limitations, as discussed in the next section. Such research demonstrates an important need for comprehensive sleep history investigations in clinical practice, which should include valuable bed partner input if possible.

**Table 2:** Specific ICSD-3 diagnostic criteria for iRBD compared with the Mayo Sleep Questionnaire questions, which can both be used by neurologists to elicit information regarding iRBD symptomology [7, 11]

ICSD-3 criteria	Mayo Sleep Questionnaire
Repeated episodes of sleep-related vocalization and/or complex motor behaviours (documented by PSG or clinical history of dream enactment behaviour) PSG recording demonstrates REM sleep without atonia (RSWA) The disturbance is not better explained by another sleep disorder, mental disorder, medication or substance abuse”	1. Have you ever seen the patient appear to “act out his/her dreams” while sleeping? (punched or flailed arms in the air, shouted or screamed). If yes, a. How many months or years has this been going on? b. Has the patient ever been injured from these behaviours (bruises, cuts, broken bones)? c. Has a bed partner ever been injured from these behaviours (bruises, blows, pulled hair)? d. Has the patient told you about dreams of being chased, attacked, or that involve defending himself/herself? e. If the patient woke up and told you about a dream, did the details of the dream match the movements made while sleeping?

## Screening questionnaires

**Fig. 1:**
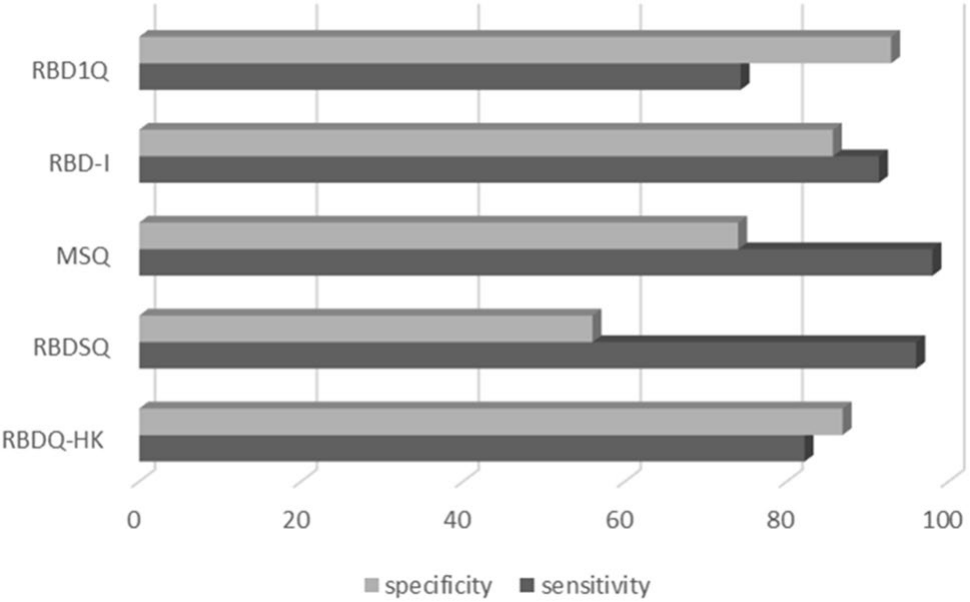
Diagnostic accuracy of iRBD screening questionnaires, separated by specificity and sensitivity values. *RBD1Q* REM Sleep Behaviour Disorder Single-Question Screen, *RBD-I* Innsbruck RBD Inventory, *MSQ* Mayo Sleep Questionnaire, *RBDSQ* REM Sleep Behaviour Disorder Screening Questionnaire, *RBDQ-HK* REM Sleep Behaviour Disorder Questionnaire Hong Kong. Adapted from [34]

One method that may aid iRBD assessment is the screening questionnaire, which can easily and quickly be administered in-person, via telehealth or online. Numerous instruments have been developed for clinical and research purposes, with variable diagnostic accuracy, dependent upon the population used for validation [34]. A recent review by Skorvanek and colleagues found that the most commonly used iRBD screening instruments comprise four specific questionnaires for iRBD, two single-item screening questions, and two generic questionnaires containing items on iRBD [34]. As summarised in Fig. 1., the sensitivity tends to be high for all screening questionnaires but differentiating iRBD from other sleep disorders is challenging, with specificity values generally quite low.

The most commonly used questionnaire in clinical populations is the REM Sleep Behaviour Disorder Screening Questionnaire (RBDSQ) [13]. This is a 10-item questionnaire examining the main clinical features of iRBD, that is self-rated by the person suspected of having iRBD and has been validated in many cross-cultural populations. It typically takes 2 min to complete and includes questions such as “My dreams frequently have an aggressive or action-packed content” and “I know that my arms or legs move when I sleep”. The RBDSQ has been found to have high diagnostic accuracy in healthy controls, with 96% sensitivity and 96% specificity. However, when including patients with other sleep disorders, the specificity drops to only 56%. This is concerning as it suggests that there is a 44% chance that sole use of the RBDSQ may mistakenly diagnose a patient with iRBD, when they have an alternate sleep disorder, such as OSA or PLMD [19, 34].

The Innsbruck RBD Inventory (RBD-I) is also widely used and contains only five self-report questions, including “Do you move out of your sleep and occasionally perform ‘‘flailing’’ or more extensive movements?” and “Have you ever injured or nearly injured yourself or your bed partner while you were sleeping?” [12]. Again, this instrument was found to have high diagnostic sensitivity (91.4) but lower specificity (85.7), revealing a propensity for false positive results. Interestingly, the only screening questionnaire to include questions specifically for bed partners, the MSQ, was shown to have sensitivity above 90% in multiple research populations (healthy controls, mild cognitive impairment, Alzheimer's disease and PD); however, specificity ranged from 36% (when compared to REM atonia index) to 95% (in older adults with 99% bed partner responses) [11, 35]. Such findings indicate that adding bed partner observations to screening data can significantly increase the detection of iRBD and it may be useful for neurologists to hand both questionnaires out in the waiting room, or after the clinic.

Validation studies show that screening questionnaires are undoubtedly useful in obtaining information about probable iRBD, yet they often result in high false-positive rates and may not be reliable in patients with cognitive impairment [36]. As such, they are not recommended as a diagnostic tool. Specifically, the ability of screening questionnaire to predict the lack of atonia during REM sleep, which is a key diagnostic criterion for iRBD, remains very low. This is in addition to their intrinsic limitation in that the patient, and even bed partner, may be completely unaware of iRBD symptoms and, therefore, unable to provide accurate information.

## Video polysomnography (vPSG)

In contrast to previous iRBD guidelines stating that either a history of injurious sleep behaviour or video-captured sleep behaviour is required for diagnosis [37], the IRBDSG now states that definitive diagnosis of iRBD requires a gold-standard, Level 1, video polysomnography (vPSG) to capture REM sleep without atonia and/or dream enactment behaviour [9]. This involves the application of several sensors to the head and body to collect physiological data during a night of sleep. Table 3 displays the American Academy of Sleep Medicine (AASM) requirements for a Level 1 vPSG [38]. Additional electromyography (EMG) data acquisition is recommended for vPSG investigating iRBD, to obtain greater muscle tone data [9, 39].

**Table 3:** American Academy of Sleep Medicine (AASM) requirements for a Level 1 vPSG, describing the electroencephalography and respiratory information obtained, as well as additional physiological data and EMG data needed for iRBD diagnosis [40]

Electroencephalography (EEG)	Gold cup electrodes positioned on the scalp to measure and record brain wave activity. This identifies sleep stages and seizure activity. The electrodes are placed according to brain regions (frontal, temporal, parietal and occipital), which is called the 10–20 system. For a standard PSG, 8 electrodes are applied in positions Fz, Cz, C3, C4, A1, A2, O1, O2 (see Fig. 2)
Respiratory	Nasal airflow pressure, mouth airflow, ribcage breathing effort, abdominal breathing effort, oximeter saturation of arterial oxygen (SaO2), intercostal electromyography (EMG) (muscle activation), genioglossus/chin EMG, abdominal EMG, tibialis anterior (TA)/leg EMG
Others	Electrocardiogram (ECG)/heart rate, body position, video/audio
Additional recommended EMG for iRBD investigations	Right and left flexor digitorum superficialis (FDS) EMG (based on IRBDSG guidelines) [9]

10–20 system placement (*Fz, Cz, C3, C4, A1, A2, O1, O2)* represents the area of the brain the electrode is reading from, i.e., frontal (F), occipital (O), central (C) and mastoid (A or M)

**Fig. 2:**
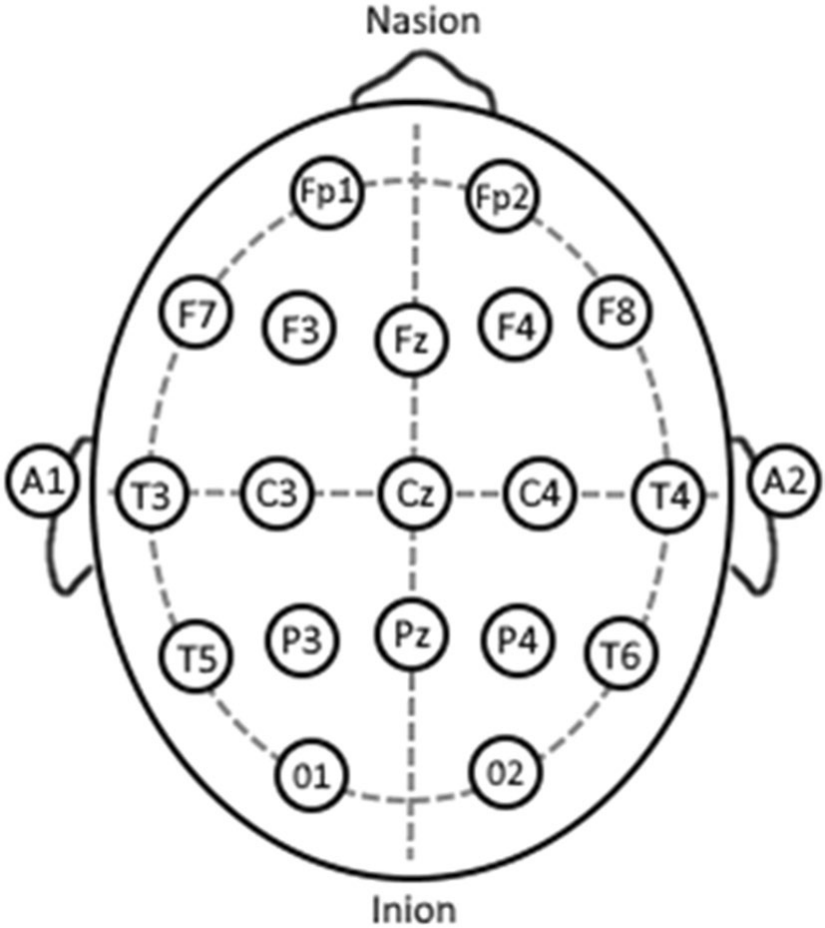
10–20 EEG Placement System. Each circle on the head represents EEG electrode placement on the patient scalp. For a standard PSG, positions *Fz, Cz, C3, C4, A1, A2, O1, O2* are used

Until recently, controversy existed around which EMG measures were most effective at delineating RSWA. Early research suggested that data from a single chin mentalis EMG trace was sufficient in identifying RSWA and thus diagnosing iRBD [41]; however, more granular EMG data collection is now recommended by sleep researchers and practitioners. The SINBAR protocol, developed by Iranzo and colleagues in 2008 states that for accurate iRBD diagnoses, EMG montage must include “mentalis in the chin, right and left flexor digitorum superficialis in the upper limbs, and right and left extensor digitorum brevis in the lower limbs” [42]. By including these five measurements of muscle activity around the mouth, and upper and lower limbs, it was found that 94.4% of motor and vocal symptoms during sleep will be detected, thus obtaining the greatest information for iRBD diagnosis [42].

To examine the usefulness of individual EMG measures, Fernandez-Arcos and colleagues analysed them separately and in combination in a sample of 49 patients with iRBD [43]. When including only isolated mentalis EMG in the evaluation, sensitivity for iRBD diagnosis was 81.6%, but when combined with upper limb muscle EMG, this increased to 91.8%. The authors suggests that 10.2% of patients may be misdiagnosed if only chin mentalis EMG is obtained from vPSG, verifying the importance of multiple EMG montages in vPSG, rather than the standard single chin mentalis EMG, to obtain maximum RSWA data and accurately diagnose iRBD.

**Fig. 3:**
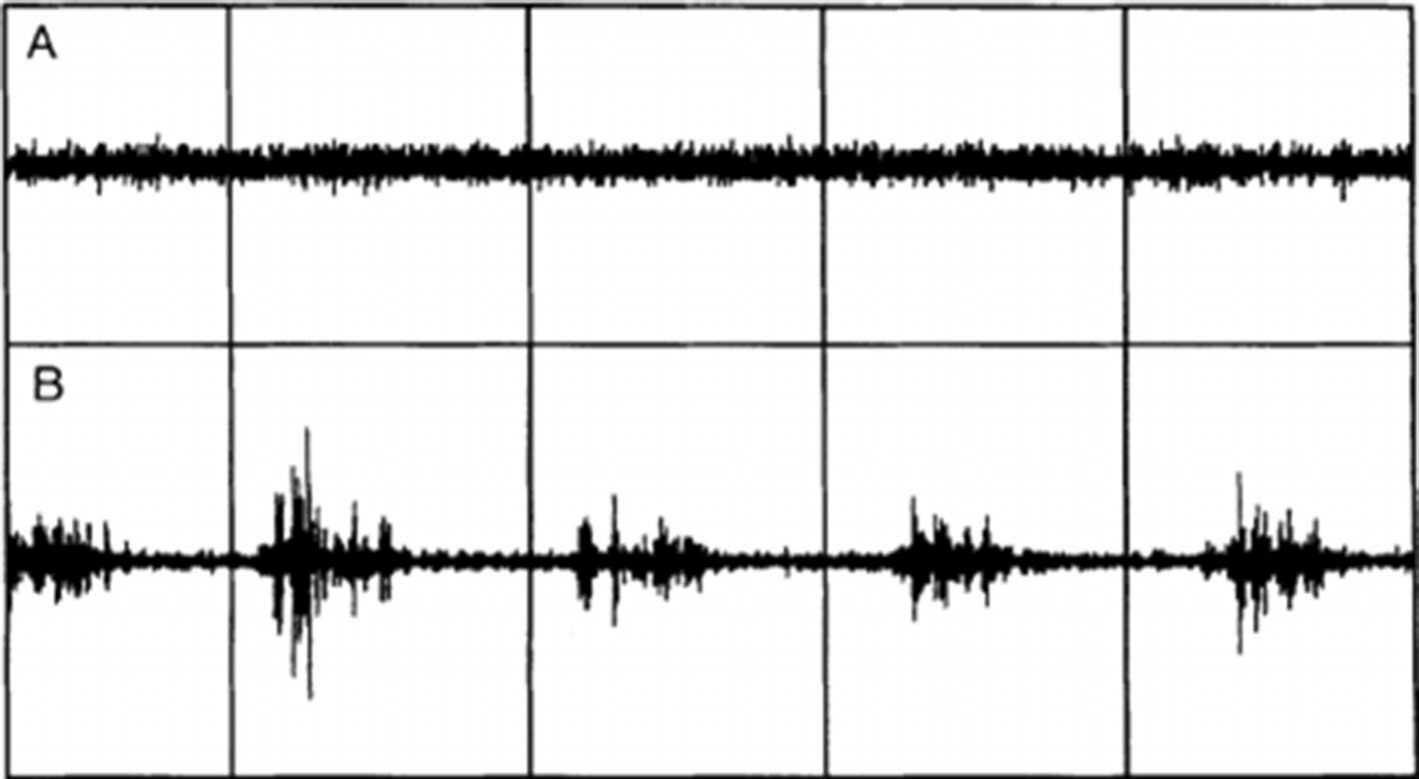
Tonic versus phasic EMG activity. A represents tonic activation and image B represents phasic activation [44]

Cesari and colleagues from the IRBDSG have since established clear international guidelines for vPSG technical requirements and RWSA quantification [9]. To accurately detect RSWA and/or motor events, vPSG should capture all physiological data as stated by the AASM, as well as additional FDS signals from the upper arms (see Table 3). They also recommend specific technical requirements for vPSG recordings, which include EMG sampling and filtering rates, video camera positioning angles, audio recording frequencies, and adjustable room temperature for individualised comfort. The IRBDSG stipulate clear guidelines for the quantification of RSWA, something which has previously been a matter of debate amongst sleep scientists. RSWA scoring rules have been mainly based upon AASM criteria; however, specific cutoff values for ‘excessive RSWA’ were ambiguous and many individual research groups were known to use slightly different rules [9]. Specific cutoff values to quantify “excessive” EMG and RSWA, are necessary to identify what is pathological. EMG activation can be quantified via sustained tonic muscle activity or excessive phasic activity. Tonic activation involves complete atonia during a REM period with a sustained increase in EMG, whereas phasic activity describes myoclonic twitches, or brief intervals of excessive EMG activity. The difference between the two can be seen in Fig. 3, where image A represents tonic activation and image B represents phasic activation.

The IRBDSG guidelines recommend that RSWA in REM sleep be quantified using phasic chin and FDS, or arm, EMG activity, analysed in 3-s epochs. Excessive chin EMG can also be calculated using any tonic or phasic activity, and at least 5 min of REM sleep is required to detect and confirm the presence of RSWA. The IRBDSG also suggest that the TA, or leg, EMG channels be included in the vPSG recording, but not for RSWA quantification, and more to rule out other sleep disorders, such as PLMD [9]. Therefore, it is clear that standard PSGs, which are most commonly available for sleep investigations, are not adequate for iRBD detection, and that neurologists may need to request additional EMG data be obtained when referring patients for vPSG. It is important to include at least chin and FDS EMG channels, as well as video recording to accurately identify episodes of RSWA, which are a core requirement for the diagnosis of iRBD.

## Limited accessibility to vPSG

The utility of vPSG is well-established in the diagnosis of iRBD, with the identification of RSWA an essential requirement. While screening questionnaires provide a general measure of likelihood of iRBD, a vPSG is required to accurately confirm the diagnosis of iRBD. However, it is challenging for most neurologists to access vPSG for their patients. For example, in Australia, the average cost of a PSG within a hospital or sleep laboratory setting is over $600, while a home-based PSG is charged at around $300. These costs are partly covered by the Australian Medicare Benefits Schedule (MBS); however, service providers often charge more than the MBS base rate, so patients need to pay the difference. Access to sleep services can also be difficult in many regional areas, with certified sleep medicine practitioners rarely available. For example, in the Australian state of Tasmania, only one lab-based sleep service currently exists, with all other services providing home-based PSG alone (without video or surface EMG for iRBD available). Many of the sleep medicine practitioners at these services are trained in respiratory sleep medicine, diagnosing mainly respiratory sleep disorders, such as obstructive sleep apnoea, with modest knowledge of less common sleep disorders and parasomnias, such as iRBD. This means that the Tasmanian population of 530,000 has access to a sole service provider of Level 1 vPSG, which is currently the only type of PSG that can accurately diagnose iRBD.

This situation is common around the world, with access to sleep investigations and treatment limited, and wait times varying from a few weeks to more than 12 months [10]. For example, in the United Kingdom, the average wait-time for a referral to a specialist is 6 months followed by a 4-month wait for a PSG, and this will cost private patients over £300. In the United States, patients can expect to pay approximately $800USD for a PSG, with a wait-time of between 3 and 9 months, depending on the area and services available [10]. Sleep service pricing appears to be highly variable and while many countries do provide government funding or rebates for sleep investigations, the out-of-pocket expense to patients is often excessive. In summary, patients throughout the world encounter substantial barriers to accessing vPSG when they have symptoms suggestive of iRBD, including lack of specialists, high financial burden and excessive wait-times. This makes it very challenging for neurologists to investigate iRBD symptoms and confirm a diagnosis in their patients.

## Emerging technologies

Within the last 10 years, increased use of basic home-based PSG (without video or additional EMG) for a variety of sleep disorders has alleviated this burden slightly. By performing PSG within the home, patients have easier access to sleep investigations in a more timely and cost-effective manner. The evidence from respiratory sleep disorders is particularly encouraging as the efficacy and reliability of home-based PSG compared with hospital/lab-based PSG has been thoroughly established as a robust method. Research shows that PSG within the home provides equivalent data to hospital/lab-based PSG in patients with sleep disordered breathing, such as obstructive sleep apnoea [45, 46], and results in good reliability, high accuracy and low-failure rate, along with greater cost-effectiveness for service providers and patients [47]. There is also strong evidence that patients achieve greater sleep quality and quantity when having a PSG in their home environment compared to a hospital or lab setting [45].

Home-based PSG may offer a potential solution for confirming a clinical diagnosis of probable iRBD. However, home PSG has not yet been validated in this clinical population and unlike respiratory disorders, iRBD diagnosis requires video confirmation of dream enactment behaviour and/or RSWA on PSG [9]. Currently, home-based PSG equipment does not routinely include video recording, as most sleep services setup patients in the office with ambulatory equipment that is worn on the body and taken home overnight. This equipment is known as a Level 2 recording (compared to a full Level 1 hospital/lab-based PSG) and does not include the complete array of data montages needed for iRBD diagnosis (such as additional EMG recording). To date, PSG equipment providers routinely manufacture home-based ambulatory equipment in line with Level 2 PSG requirements, making it difficult to assess iRBD within the home as it does not normally allow for extra EMG data collection or video recording. There remains a need for PSG equipment that can be adapted to record Level 1 iRBD studies within the home.

In recognition of the difficulties for most neurologists in access to lab and home-based PSG, and the growing need to make accurate early diagnoses of iRBD for clinical trials, recent research has focused on evaluating new computer-based technologies to assist in diagnosis of iRBD. These offer lower costs, easier accessibility, and potential widespread use via wireless technology, mobile phone applications and telehealth, making the future of diagnostic resources limitless.

## Actigraphy

**Fig. 4:**
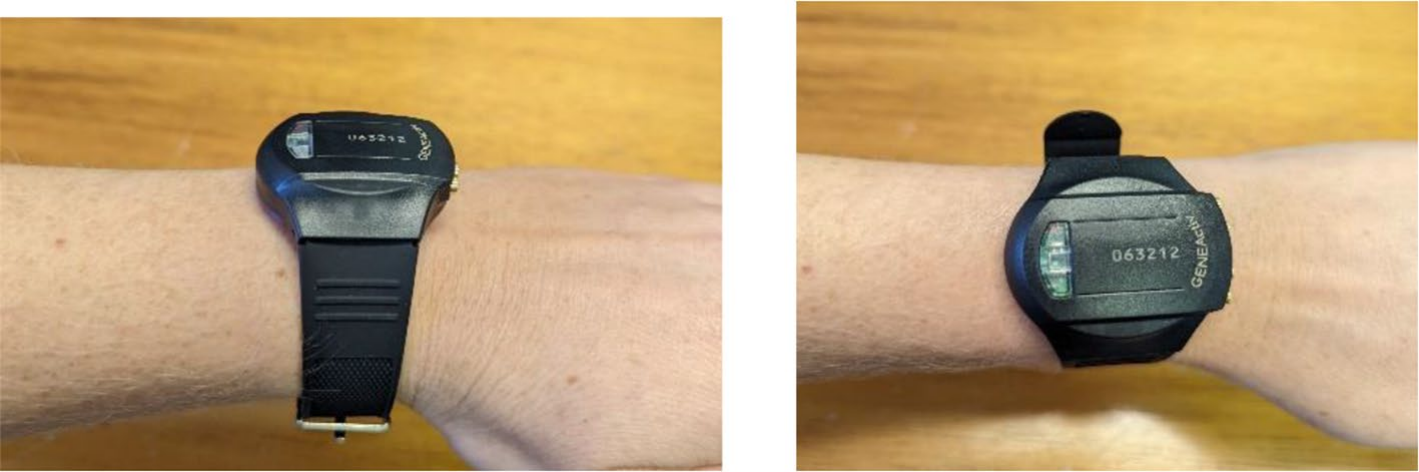
Actigraphy wrist device

Improvement in movement analysis, artificial intelligence (AI) technologies and general acceptance of watch style devices has generated an unconventional and less expensive avenue of sleep measurement with the creation of actigraphy [48]. Actigraphy devices cost between 200€ and 1,100€ EUR ($300 and $1700 AUD) depending on models, and are typically worn on the wrist, recording movement (with some recording light and temperature data as well) over a 24-h period (see Fig. 4).

Computerised algorithms are then used to estimate sleep parameters, such as total sleep time, sleep percentage, and wake after sleep onset, all of which can aid in the assessment of sleep disorders [49]. Actigraphy provides a good objective measurement of sleep patterns over time, as they are non-invasive devices and can be worn for multiple days or weeks at a time.

They are particularly useful in identifying sleep–wake disturbances and circadian rhythm disorders but cannot capture all other measures obtained from PSG. The first study to investigate the use of actigraphy in RBD diagnosis was published in 2010 by Naismith and colleagues [50]. A sample of patients with PD was screened using the RBDSQ and then wore a wrist actigraphy watch over 14 days. Out of 22 patients, 13 screened positive on the questionnaire, suggesting probable RBD. These patients showed a significantly greater number of overnight awakenings on actigraphy compared to patients who screened negatively on the RBDSQ, confirming that those with probable RBD have clearly different nocturnal disturbance patterns, which can be captured via actigraphy. In 2014, Louter and colleagues also investigated the accuracy of actigraphy to detect RBD in 45 patients with PD [51]. All patients completed one night of vPSG (in a sleep laboratory) and eight nights of actigraphy at home, starting on the night of the vPSG. Total sleep time, sleep latency, sleep efficiency, and number and length of wake bouts were measured using actigraphy. Similar to Naismith and colleagues, results showed that the only significant correlation between vPSG and actigraphy was the number of wake bouts overnight, which was found to be 30% greater in patients with confirmed RBD than those without. This suggests that actigraphy has high specificity and that number of awakenings overnight may be predictive of RBD in patients with PD. However, Louter and colleagues did find differences in total sleep time and sleep efficiency between actigraphy and vPSG recordings, indicating notable limitations in the accuracy of actigraphy to discern RBD movements from other activities overnight, such as tremor or dyskinesias [51].

In 2018, Stefani and colleagues evaluated the use of actigraphy to screen for iRBD in 90 individuals, 70 of whom had sleep disorders with notable movements, (iRBD, sleep apnoea and restless legs syndrome) and 20 patients without motor manifestations during sleep [52]. They all completed six validated RBD screening questionnaires and underwent one night of vPSG and 2 weeks of actigraphy. The actigraphy data was analysed quantitatively using computerised software and visually by seven blinded raters (sleep medicine experts) who had access to additional clinical information. Using quantitative analysis, the actigraphy method distinguished iRBD from control participants, with activity score, activity index, and short burst inactivity index significantly higher in patients with iRBD than controls; however, data patterns were not clearly differentiated between those with iRBD and those with other sleep movement disorders. The visual analysis of actigraphy data by experts resulted in a 67–84% accuracy of iRBD identification, and when used in conjunction with clinical data this increased to an accuracy rate of 86.4%.

Further investigations by this research group in 2020 looked more closely at objective rest-activity cycles of iRBD to assist in diagnosis [53]. Using similar methodology, 2 weeks of actigraphy was compared against vPSG in multiple patient samples (iRBD, restless leg syndrome, sleep apnoea and healthy controls). A new index was formulated (*I* < *O*) which expresses the relationship between nocturnal and diurnal motor intensity over a 24-h period, using non-parametric data including consistency of daily motor activity and the amplitude of rest-activity patterns. Actigraphy data resulted in statistically significant differences in estimated sleep efficiency, wake after sleep onset, prolonged activity bouts, naps, and *I* < *O* between groups. Patients with iRBD showed lower sleep efficiency and higher wake after sleep onset than patients with restless leg syndrome. They also shower greater rates of prolonged activity bouts, more naps and lower *I* < *O* than all other groups. Overall, the *I* < *O* index was found to accurately distinguish between patients with iRBD and controls, but also between iRBD, restless leg syndrome and sleep apnoea.

Taken together, these findings indicate that actigraphy shows strong potential as an additional tool in the detection of iRBD, especially when detailed clinical information is available. Discernible sleep–wake patterns appear to exist in iRBD which may help clinicians identify patients with possible iRBD and add further confidence to the clinical diagnosis of probable iRBD. However, at this stage, diagnosis from actigraphy should still be viewed cautiously considering the early stage research and recognising that other sleep disorders may manifest with similar patterns, such as periodic limb movement disorder, which have not yet been investigated with this technology.

## Technologies under development

With billions of people having access to smartphones worldwide [54], there has been considerable interest in developing apps to precisely detect different stages of sleep. Advancing smartphone applications provide a perfect opportunity to assess sleep on a large scale, with low costs and the possibility of longer term sleep monitoring [55]. One of the first studies to examine the utility of a smartphone application was by Bhat and colleagues in 2015, who compared the output of a smart phone sleep application to a laboratory-based PSG [56]. The application aimed to measure sleep patterns by detecting body movements through the smartphone’s in-built accelerometer placed on the patient’s bed. Results showed that the application was highly effective (85.9%) at identifying sleep and wakefulness overnight, but its specificity was poor, overpredicting sleep 50% of the time. No correlations were found between the PSG and sleep application in sleep efficiency, sleep latency, or light and deep sleep percentage overnight [56], suggesting that a singular smart phone application relying on accelerometer data is likely to be ineffective at identifying sleep accurately, let alone a sleep disorder.

More recent research indicates that combining smart phone applications with additional measuring devices may present a more accurate route to sleep disorder detection. Xu and colleagues reviewed the scientific quality of sleep applications available in China in comparison to PSG [57]. After reviewing a total of 2,369 applications, they found that those which connected to other devices, such as smartwatches, had a greater scientific score (based on data similar to that obtained via PSG) than those based solely on smartphone data. However, this may be dependent upon the type of additional device. A 2016 study by Toon and colleagues compared sleep parameters using a commercial wrist accelerometer, smart phone accelerometer application and wrist actigraphy against PSG in a sample of children and adolescents with suspected sleep disordered breathing [58]. Their results indicated that data on total sleep time, wake after sleep onset and sleep efficiency obtained from the commercial accelerometer and wrist actigraphy were comparable to PSG, such that they could assess sleep and wake with an accuracy rate of 86%. Unsurprisingly, the smartphone application alone did not correlate well with data from PSG or wrist devices, significantly overestimating total sleep time and sleep efficiency, suggesting that accelerometer based smart phones applications may not be suitable to assess sleep parameters or disorders.

Further research using smart phone technology with a different type of device investigated sleep parameters using an under-the-mattress piezoelectric sensor [59]. This sensor, when placed under a person's mattress, sends data to a specifically programmed smart phone application, which has been validated in the measurement of body movements, heart rate and respiration [60]. Tal and colleagues’ 2017 study showed that when compared to PSG, this contact-free system found similar percentages of total sleep time, wakefulness, REM sleep, NREM sleep, and over 90% accuracy in continuous measurement of heart rate and respiration. A more recent study based on alternate smart phone technology may also improve the outlook on these smart phone devices. In 2019, Lyon and colleagues assessed the usefulness of another contact-free sleep monitoring system using sonar technology [61]. By placing a smartphone equipped with a custom sonar enabled application, the researchers were able to measure respiration and movement overnight. With a focus on sleep disordered breathing, results showed that sonar obtained data had a sensitivity of 94% and specificity of 97% when identifying respiratory related events, such that two thirds of respiratory events (apnoeas) corresponded with PSG derived severity. The authors suggest that sonar technology is cost effective, easy to use and can provide reliable and accurate detection of SDB, making it a potentially valuable clinical tool in the identification of other sleep disorders as well. Taken together, these studies provide evidence of the need for further research into alternate technologies for the measurement of iRBD and other sleep disorders, as it may be that combinations of the devices produce the best alternative to PSG. It is also important to note that none of the new wearable technologies described in this section are compliant with accepted diagnostic criteria. However, the IRBDSG state that “In the future, at-home technologies might help to identify these conditions [RBD and prodromal RBD] in the general population, as well as to monitor their evolution over time. Actigraphy, motion activated video-recording, automatic analysis of 3-D videos, and light and practical devices to record sleep, are promising tools that should be further investigated in the future and compared to gold standard v-PSG" [9].

## Conclusions

It can be challenging to accurately diagnose iRBD. There is typically a substantial delay before a neurologist is consulted: many people are simply not aware of their nocturnal signs and the symptoms described can mimic or masquerade other sleep disorders. Furthermore, many GPs or other ‘first consulted’ practitioners will not recognise the significance of the symptoms or consider the diagnosis of iRBD as it is a lesser-known condition. For neurologists, and other clinicians diagnosing iRBD, it is paramount to interview the bed partner, but even with a collateral history from an observant bed partner, it can be challenging to discriminate iRBD from similar clinical manifestations of disorders, such as OSA or PLMD. Questionnaires may support clinicians to make a ‘probable diagnosis’ but should not be used as a sole diagnostic tool. Ultimately a gold standard diagnosis requires vPSG, which is often difficult to access and costly.

There is an urgent need for vPSG availability within the home, to increase accessibility and improve early diagnosis in this high-risk population. There are encouraging results from respiratory sleep disorders diagnosed through home PSG, but no research studies have yet evaluated home vPSG for the diagnosis of iRBD. Without easier access to reliable and validated vPSG, this population cannot readily access clinical trials or risk modification studies and our abilities to better understand the disorder through observational studies is severely hindered. Recent advances in technologies such as actigraphy and contactless devices presents an exciting and much more accessible way of detecting iRBD; however, there have been limited studies so far and further research is needed.

With people living longer around the world, the number of people at risk of neurodegenerative diseases is increasing dramatically. Detecting iRBD presents a critical opportunity to intervene early, through drugs or risk modification, to modify its trajectory or even prevent the development of future neurodegenerative diseases. Increasing interest in pharmaceutical clinical trials for iRBD further increases the importance of diagnosis and neurologists play a central role in early, accurate diagnosis of this disorder.

## References

[1] Carskadon MA, Dement WC, Kryger MH, Roth T, Dement WC (2011). Monitoring and staging human sleep. Principles and practice of sleep medicine.

[2] Iranzo A (2018). The REM sleep circuit and how its impairment leads to REM sleep behavior disorder. Cell Tissue Res.

[3] Schenck CH, Lee SA, Cramer Bornemann MA, Mahowald MW (2009). Potentially lethal behaviors associated with rapid eye movement sleep behavior disorder: review of the literature and forensic implications. J Forensic Sci.

[4] Iranzo A, Santamaria J (2005). Severe obstructive sleep apnea/hypopnea mimicking REM sleep behavior disorder. Sleep.

[5] Postuma RB (2019). Risk and predictors of dementia and Parkinsonism in idiopathic REM sleep behaviour disorder: a multicentre study. Brain.

[6] Schenck CH (2013). Delayed emergence of a Parkinsonian disorder or dementia in 81% of older men initially diagnosed with idiopathic rapid eye movement sleep behavior disorder: a 16-year update on a previously reported series. Sleep Med.

[7] Högl B, Stefani A, Videnovic A (2018). Idiopathic REM sleep behaviour disorder and neurodegeneration—an update. Nat Rev Neurol.

[8] Hogl B, Stefani A (2017). REM sleep behavior disorder (RBD): update on diagnosis and treatment. Somnologie (Berl).

[9] Cesari M, Heidbreder A, St-Louis EK (2022). Video-polysomnography procedures for diagnosis of rapid eye movement sleep behavior disorder (RBD) and the identification of its prodromal stages: guidelines from the International RBD Study Group. Sleep.

[10] Flemons WW (2004). Access to diagnosis and treatment of patients with suspected sleep apnea. Am J Respir Crit Care Med.

[11] Boeve BF (2013). Validation of the Mayo Sleep Questionnaire to screen for REM sleep behavior disorder in a community-based sample. J Clin Sleep Med.

[12] Frauscher B (2012). Validation of the Innsbruck REM Sleep Behavior Disorder Inventory. Mov Disord.

[13] Stiasny-Kolster K, Mayer G, Schafer S (2007). The REM Sleep Behavior Disorder Screening Questionnaire—a new diagnostic instrument. Mov Disord.

[14] Stiasny-Kolster K, Sixel-Döring F, Trenkwalder C (2015). Diagnostic value of the REM sleep behavior disorder screening questionnaire in Parkinson's disease. Sleep Med.

[15] Stefani A (2018). Screening for idiopathic REM sleep behavior disorder: usefulness of actigraphy. Sleep.

[16] Matar E, McCarter SJ, St Louis EK, Lews SJG (2021). Current concepts and controversies in the management of REM sleep behavior disorder. Neurotherapeutics.

[17] White C (2012). Diagnostic delay in REM sleep behavior disorder (RBD). J Clin Sleep Med.

[18] Saleem AH, Al Rashed FA, Alkharboush GA (2017). Primary care physicians' knowledge of sleep medicine and barriers to transfer of patients with sleep disorders. A cross-sectional study. Saudi Med J.

[19] Gaig C, Iranzo A, Pujol M (2017). Periodic limb movements during sleep mimicking REM sleep behavior disorder: a new form of periodic limb movement disorder. Sleep.

[20] Tan L, Zhou J, Yang L (2017). Duloxetine-induced rapid eye movement sleep behavior disorder: a case report. BMC Psychiatry.

[21] Kierlin L, Littner MR (2011). Parasomnias and antidepressant therapy: a review of the literature. Front Psychiatry.

[22] Palma JA, Fernandez-Cordon C, Coon EA (2015). Prevalence of REM sleep behavior disorder in multiple system atrophy: a multicenter study and meta-analysis. Clin Auton Res.

[23] Frauscher B, Jennum P, Ju YE, Postuma RB (2014). Comorbidity and medication in REM sleep behavior disorder: a multicenter case-control study. Neurology.

[24] Enriquez-Marulanda A, Quintana-Pena V, Takeuchi Y, Quinones J (2018). Case report: rapid eye movement sleep behavior disorder as the first manifestation of multiple sclerosis: a case report and literature review. Int J MS Care.

[25] Tang WK, Hermann DM, Chen YK (2014). Brainstem infarcts predict REM sleep behavior disorder in acute ischemic stroke. BMC Neurol.

[26] Coeytaux A, Wong K, Grunstein R, Lewis SJG (2013). REM sleep behaviour disorder—more than just a parasomnia. Aust Fam Physician.

[27] Giannini G, Provini F, Cortelli P, Calandra-Buonaura G (2021). REM sleep behaviour disorder in multiple system atrophy: from prodromal to progression of disease. Front Neurol.

[28] Gieselmann A, Ait Aoudia M, Carr M (2019). Aetiology and treatment of nightmare disorder: State of the art and future perspectives. J Sleep Res.

[29] Frauscher B, Gschliesser V, Brandauer, (2010). REM sleep behavior disorder in 703 sleep-disorder patients: the importance of eliciting a comprehensive sleep history. Sleep Med.

[30] Roguski A, Rayment D, Whone AL, Jones MW, Rolinski M (2020). A neurologist's guide to REM sleep behavior disorder. Front Neurol.

[31] Vignatelli L (2003). Interobserver reliability of ICSD-R criteria for REM sleep behaviour disorder. J Sleep Res.

[32] McCarter SJ, Louis EKS, Boswell CL (2014). Factors associated with injury in REM sleep behavior disorder. Sleep Med.

[33] Rodriguez JC, Dzierzewski JM, Alessi CA (2015). Sleep problems in the elderly. Med Clin N Am.

[34] Skorvanek M, Feketeova E, Kurtis MM, Rusz J, Sonka K (2018). Accuracy of rating scales and clinical measures for screening of rapid eye movement sleep behavior disorder and for predicting conversion to parkinson's disease and other synucleinopathies. Front Neurol.

[35] Bolitho SJ, Naismith SL, Terpening Z (2014). Investigating rapid eye movement sleep without atonia in Parkinson’s disease using the rapid eye movement sleep behavior disorder screening questionnaire. Mov Disord.

[36] Wilson G, Terpening Z, Wong K, Grunstein (2014). Screening for sleep apnoea in mild cognitive impairment: the utility of the multivariable Apnoea Prediction Index. Sleep Disord.

[37] Aurora RN, Zak RS, Maganti RK (2010). Best practice guide for the treatment of REM sleep behavior disorder (RBD). J Clin Sleep Med.

[38] Ruehland WR, O’Donoghue FJ, Pierce RJ (2011). The 2007 AASM recommendations for EEG electrode placement in polysomnography: impact on sleep and cortical arousal scoring. Sleep.

[39] Frauscher B, Iranzo A, Gaig C (2012). Normative EMG values during REM sleep for the diagnosis of REM sleep behavior disorder. Sleep.

[40] . Berry RB et al. (2020) The AASM Manual for the Scoring of Sleep and Associated Events: Rules, Terminology and Technical Specifications: Version 2.6. American Academy of Sleep Medicine, Darien

[41] Shokrollahi M, Krishnan S, Kumar D, Arjunan S (2012) Chin EMG analysis for REM sleep behavior disorders. In: biosignals and biorobotics conference: biosignals and robotics for better and safer living (BRC)

[42] Iranzo A, Frauscher B, Santos H, et, (2011). Usefulness of the SINBAR electromyographic montage to detect the motor and vocal manifestations occurring in REM sleep behavior disorder. Sleep Med.

[43] Fernandez-Arcos A, Iranzo A, Serradell M (2017). Diagnostic value of isolated mentalis versus mentalis plus upper limb electromyography in idiopathic REM sleep behavior disorder patients eventually developing a neurodegenerative syndrome. Sleep.

[44] Rahman MQ, Kingshott RN, Wraith WH, Adams WH, Drummond GB (2001). Association of airway obstruction, sleep, and phasic abdominal muscle activity after upper abdominal surgery. Br J Anaesth.

[45] Bruyneel M, Ninane V (2014). Unattended home-based polysomnography for sleep disordered breathing: current concepts and perspectives. Sleep Med Rev.

[46] Bruyneel M, Sanida C, Art G (2011). Sleep efficiency during sleep studies: results of a prospective study comparing home-based and in-hospital polysomnography. J Sleep Res.

[47] Banhiran W, Chotinaiwattarakul W, Chongkolwatana C, Metheetrairut C (2014). Home-based diagnosis of obstructive sleep apnea by polysomnography type 2: accuracy, reliability, and feasibility. Sleep Breath.

[48] Smith MT, McCrae CS, Cheung J (2018). Use of actigraphy for the evaluation of sleep disorders and circadian rhythm sleep-wake disorders: an American Academy of Sleep Medicine systematic review, meta-analysis, and GRADE assessment. J Clin Sleep Med.

[49] Martin JL, Hakim AD (2011). Wrist actigraphy. Chest.

[50] Naismith SL, Rogers NL, Mackenzie J, Hickie IB, Lewis SJG (2010). The relationship between actigraphically defined sleep disturbance and REM sleep behaviour disorder in Parkinson's disease. Clin Neurol Neurosurg.

[51] Louter M, Arends J, Bloem BR, Overeem S (2014). Actigraphy as a diagnostic aid for REM sleep behavior disorder in Parkinson's disease. BMC Neurol.

[52] Stefani A, Heidbreder A, Brandauer E (2018). Screening for idiopathic REM sleep behavior disorder: usefulness of actigraphy. Sleep.

[53] Filardi M, Stefani A, Holzknecht E, Pizza F, Plazzi G, Hogl B (2020). Objective rest-activity cycle analysis by actigraphy identifies isolated rapid eye movement sleep behavior disorder. Eur J Neurol.

[54] Poushter J (2016). Smartphone ownership and internet usage continues to climb in emerging economies. Pew Res Center.

[55] Fino E, Mazzetti M (2019). Monitoring healthy and disturbed sleep through smartphone applications: a review of experimental evidence. Sleep Breath.

[56] Bhat S, Ferraris A, Gupta D (2015). Is there a clinical role for smartphone sleep apps? Comparison of sleep cycle detection by a smartphone application to polysomnography. J Clin Sleep Med.

[57] Xu ZF, Luo X, Shi J, Lai Y (2019). Quality analysis of smart phone sleep apps in China: can apps be used to conveniently screen for obstructive sleep apnea at home?. BMC Med Inform Decis Mak.

[58] Toon E, Davey MJ, Hollis SL (2016). Comparison of commercial wrist-based and smartphone accelerometers, actigraphy, and PSG in a clinical cohort of children and adolescents. J Clin Sleep Med.

[59] Tal A, Shinar Z, Shaki D, Codish S, Goldbart A (2017). Validation of contact-free sleep monitoring device with comparison to polysomnography. J Clin Sleep Med.

[60] Ben-Ari J, Zimlichman E, Adi N, Sorkine P (2010). Contactless respiratory and heart rate monitoring: validation of an innovative tool. J Med Eng Technol.

[61] Lyon G, Tiron R, Zaffaroni A (2019) Detection of sleep apnea using sonar smartphone technology. In: Conference proceedings: Annual International Conference of the IEEE Engineering in Medicine and Biology Society. 10.1109/EMBC.2019.8857836 10.1109/EMBC.2019.8857836 31947494

